# Expression of Tenascin-C Is Upregulated in the Early Stages of Radiation Pneumonitis/Fibrosis in a Novel Mouse Model

**DOI:** 10.3390/cimb46090575

**Published:** 2024-09-01

**Authors:** Kazuki Omori, Akinori Takada, Yutaka Toyomasu, Isao Tawara, Chihiro Shintoku, Kyoko Imanaka-Yoshida, Hajime Sakuma, Yoshihito Nomoto

**Affiliations:** 1 Department of Radiology, Mie University Hospital, Tsu 514-8507, Mie, Japan; k-omori@med.mie-u.ac.jp (K.O.); y-toyomasu@med.mie-u.ac.jp (Y.T.); sakuma.mie@gmail.com (H.S.); nomoto-y@med.mie-u.ac.jp (Y.N.); 2 Department of Hematology and Oncology, Mie University Hospital, Tsu 514-8507, Mie, Japan; itawara@med.mie-u.ac.jp; 3 Department of Pathology and Matrix Biology, Mie University Graduate School of Medicine, Tsu 514-8507, Mie, Japan; 318047@m.mie-u.ac.jp (C.S.); imanaka@med.mie-u.ac.jp (K.I.-Y.)

**Keywords:** tenascin-C, extracellular matrix glycoprotein, radiation pneumonia, radiation fibrosis, stereotactic body radiotherapy, radiation-induced lung damage

## Abstract

The lung is a major dose-limiting organ for radiation therapy (RT) for cancer in the thoracic region, and the clarification of radiation-induced lung damage (RILD) is important. However, there have been few reports containing a detailed comparison of radiographic images with the pathological findings of radiation pneumonitis (RP)/radiation fibrosis (RF). We recently reported the upregulated expression of tenascin-C (TNC), an inflammation-associated extracellular matrix molecule, in surgically resected lung tissue, and elevated serum levels were elevated in a RILD patient. Therefore, we have developed a novel mouse model of partial lung irradiation and studied it with special attention paid to the computed tomography (CT) images and immunohistological findings. The right lungs of mice (BALB/c) were irradiated locally at 30 Gy/1fr, and the following two groups were created. In Group 1, sequential CT was performed to confirm the time-dependent changes in RILD. In Group 2, the CT images and histopathological findings of the lung were compared. RP findings were detected histologically at 16 weeks after irradiation; they were also observed on the CT images from 20 weeks. The immunostaining of TNC was observed before the appearance of RP on the CT images. The findings suggest that TNC could be an inflammatory marker preceding lung fibrosis.

## 1. Introduction

The lung is a major dose-limiting organ for radiation therapy (RT) for cancer in the thoracic region. Recently, high-dose per fraction hypofractionated radiotherapy, including stereotactic body radiotherapy (SBRT), has emerged as a useful modality for lung cancers. In SBRT, imaging scans are used to guide the radiation. Radiation is delivered intensively to the target area from multiple directions, sparing the nearby healthy lung tissue. Radiation-induced lung damage (RILD) is classified into acute radiation pneumonitis (RP) and late-occurring radiation fibrosis (RF). RP typically presents 1 to 6 months after RT. It advances to RF over several months to years. In some cases, RILD can become fatal [1,2,3]. Radiographically, computed tomography (CT) findings of RP show diffuse or patchy ground-glass opacities or consolidation [3]. Subsequently, RP changes to RF, with chronic consolidation, linear scarring, lung contractions, traction bronchiectasis, and the deviation of the mediastinum toward the irradiated region [1]. In experimental studies, there have been reports of entire lung or hemi-lung irradiation in mice [2,4,5,6,7]. However, there have been few reports containing a detailed comparison of radiographic images with the pathological findings of RILD.

Previous studies have shown that vascular injury and the coagulation cascade, cellular adhesion molecules, proinflammatory and profibrotic cytokines, and oxidative stress play important roles in the development of RILD [8]. In the acute phase of RILD, damage to DNA or cytoplasmic organelles triggers may trigger intracellular signaling, leading to altered gene expression and the immediate release of growth factors such as transforming growth factor β (TGF-β), platelet-derived growth factor, and interleukin 1. Additionally, the ionization of water molecules may generate reactive oxygen species (ROS). ROS cause cell loss, edema of the alveolar walls, increased vascular permeability, and protein exudation into the alveolar space, which reduces the alveolar septa and vascular integrity, leading to the apoptosis of alveolar type-I pneumocytes [9]. In the late phase of RILD, TGF-β produced by activated immune cells activates multiple signaling cascades, including the small mother against decapentaplegic 2/3, mitogen-activated protein kinase, and extracellular signal-regulated kinase signaling pathways. TGF-β stimulates the expression of fibrillar collagens and fibronectin by fibroblasts in the interstitial space. Additionally, lung architecture remodeling results in the increased stiffness and thickening of the lung parenchyma due to the overproduction of extracellular matrix proteins, substantially decreasing gas exchange [10]. However, the detailed molecular mechanisms of RILD are not well understood, and no specific molecular markers or therapeutic agents have been developed.

Tenascin-C (TNC) is a large hexametric extracellular matrix glycoprotein that is sparsely expressed in normal tissue but transiently expressed at specific sites during inflammation, wound healing, and cancer invasion [11,12]. Increasing evidence has shown the diverse functions of TNC, particularly its proinflammatory [13,14] and profibrotic functions [15,16]. Furthermore, TNC is known to be one of the early markers of inflammation and progressing fibrosis in various tissue types, such as the heart [11,17,18,19]. It has been reported that TGF-β-mediated fibrosis in bleomycin-induced lung injury was suppressed in TNC-deficient mice [20]. In humans, TNC was almost undetected in normal lungs but significantly upregulated in fibrotic lungs, such as in idiopathic pulmonary fibrosis and chronic hypersensitivity pneumonitis [21]. After the irradiation of the lungs, inflammation occurs in the lung tissue, and various factors related to tissue repair are activated. TNC might be one of these factors. Therefore, we hypothesized that TNC contributes to the progression of inflammation and tissue remodeling during the development of RILD. Recently, we reported TNC expression in the surgically resected lung tissue of a patient with RP and the elevation of the serum levels after radiation [22]. These findings suggest that TNC could serve as a potential biomarker and therapeutic target for RILD. In this study, we created a new mouse model of RP/RF via partial lung irradiation. We sequentially examined the CT images and compared them with the pathological findings to elucidate the relationship between RP/RF and TNC.

## 2. Materials and Methods

### 2.1. Mice and Pb Shielding Device

**Figure 1:**
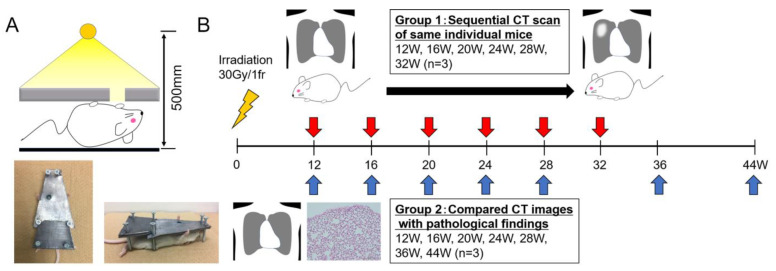
(**A**) The radiation shielding device: this device consists of a 3-mm-thick Pb and a hole with a diameter of 12 mm around the right lung of the mouse. Anesthetized mice were covered with the device and irradiated from the ventral side in the supine position. (**B**) Study design: after the irradiation of 12-week-old BALB/c mice at 30 Gy/1fr, the mice were categorized into two groups.

Female BALB/c mice, aged 12 weeks, were purchased from Charles River Laboratories Japan, Inc. (Yokohama, Kanagawa, Japan). All mice had free access to a standard diet and water and were housed under a 12 h dark–light cycle at 22 ± 1 °C. The animal research committee of our institution approved the animal experimental procedures (approved #30-15). We created an original radiation shielding device composed of a Pb block, which was trapezoidal, with a short base of 1 cm, a long base of 5 cm, a height of 7.5 cm, a thickness of 3 mm, and a hole 12 mm in diameter around the right lungs of the mice. Additionally, the height of the device could be adjusted from 0 cm to 2.5 cm using screws to avoid compressing the mice. The device enabled us to partially irradiate the right lungs of the mice (Figure 1A).

### 2.2. Irradiation and CT Scans

For irradiation, the mice were anesthetized with an intraperitoneal injection of butorphanol tartrate (5 mg/kg), medetomidine hydrochloride (0.3 mg/kg), and midazolam (4 mg/kg) and placed under the Pb shielding device. Subsequently, the right lung of each mouse was exposed to X-rays using CAX-150-20 (Chubu Medical Co., Ltd., Yokkaichi, Mie, Japan). Irradiation was performed at 150 kV and 20 mA through a 1 mm aluminum and 0.1 mm copper filter, with ionizing radiation of 30 Gy at 65.8 cGy/min from a single ventral field. The irradiation was performed at a distance of 500 mm from the X-ray focus to the bed table. Briefly, the conditions for irradiation are described in Figure 1A.

After irradiation, the mice were randomly categorized into the following two groups: Group 1 (three mice underwent sequential CT to confirm the time-dependent image changes in RP/RF at 12, 16, 20, 24, 28, and 32 weeks post-irradiation; n = 3) and Group 2 (CT images were compared with the histopathological findings of the lung (12, 16, 20, 24, 28, 36, and 44 weeks post-irradiation; n = 3)) (Figure 1B).

For micro-CT imaging, the mice were anesthetized via the inhalation of 1.5% isoflurane. Subsequently, CT images were acquired using three-dimensional micro-CT (R_mCT; Rigaku Co., Ltd., Akishima, Tokyo, Japan), with a tube voltage and current of 60 kV and 100 mA, respectively. The exposure time was 17 s, and the images were reconstructed at a 400 μm thickness by obtaining end-expiratory images using a respiratory-gated imaging technique.

In Group 1, sequential computed tomography (CT) scans were obtained for the same mice at 12, 16, 20, 24, 28, and 32 weeks after irradiation. In this group, to eliminate individual differences in observations, we performed sedation and the CT evaluation of RILD on the same mouse each time.

In Group 2, the CT images and pathological findings were compared at 12, 16, 20, 24, 28, 36, and 44 weeks after irradiation. In this group, to confirm the CT findings of RILD pathologically, we performed autopsies at each time point.

### 2.3. Histopathological Analysis of Lung Tissue

The mice were euthanized with carbon dioxide, and the lung lumen was filled with 4% formalin in phosphate-buffered saline (PBS; pH: 7.0–7.4) via trachea cannulation with a 22-gauge needle. Subsequently, the lungs were removed and fixed in 4% formalin in PBS overnight before being immersed in paraffin. Next, we prepared and stained 5 μm sections with hematoxylin and eosin (H-E) or Elastica Sirius Red (SR). SR staining is a histological technique used to differentiate between and visualize elastic fibers and collagen fibers in tissue sections. As described previously, collagen fiber formation was observed in SR-stained slides under polarized light microscopy with appropriate band-pass filters [23]. Additionally, collagen-type-I-rich thick and type-III-rich thin fibers were identified using polarization microscopy as yellow–red and green fibers, respectively. As previously described, the sections were immunohistochemically stained with rabbit polyclonal anti-TNC antibodies (dilution 1:100) after antigen retrieval (0.05% pepsin, 37 °C for 10 min) to evaluate TNC expression [24]. For the cleavage of peptides masking antigenic epitopes of TNC, the sections were incubated in 0.4% pepsin (1:60,000; Sigma Chemical Corporation, St. Louis, MO, USA) in 0.01 N HCl for 10 min at 37 °C to retrieve the antigens. Macrophage marker F4/80 (rat monoclonal antibody ab6640, ABCAM, Cambridge, UK) and T lymphocyte marker CD3 (rabbit polyclonal antibody ab5690, ABCAM) were immunostained according to the manufacturing datasheets, as previously described [14].

### 2.4. Quantification of TNC-Expressing Regions

The deposition of TNC at 12, 16, 20, 24, 28, 36, and 44 weeks after irradiation (n = 3 for each point) was quantified. Two consecutive sections of the lung from each mouse were prepared to measure the areas of the lung and TNC-expressing regions. These areas were measured with ImageJ version 1.54 (NIH, Bethesda, MD, USA) (Java 1.8.0_322). The lung areas were measured by delineating the lung region as a region of interest on H-E staining images. Images of immunohistochemically stained TNC were converted to 16-bit monochrome photographs and thresholded using ImageJ’s “MaxEntropy” algorithm to delineate TNC-positive regions, and the area of the TNC-expressing region was measured. Finally, the area ratio of the TNC-expressing region (TNC-positive/entire lung) was calculated.

## 3. Results

### 3.1. Sequential CT Image Changes in Focally Irradiated Lung of Mouse with 30 Gy/1fr

**Figure 2:**
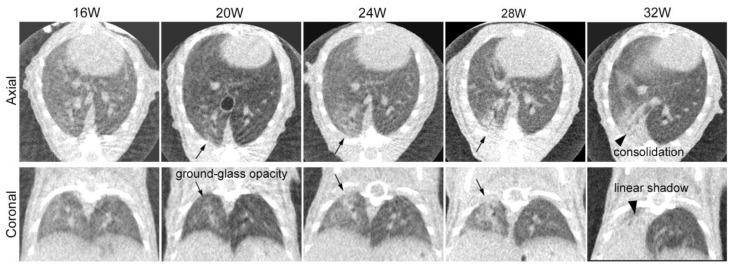
Sequential computed tomography (CT) axial and coronal images of a mouse after partial right lung irradiation at 30 Gy/1fr: CT scans were sequentially obtained for the same mice to confirm the time-dependent changes in the irradiated lung. We found no change until 16 weeks after irradiation. Findings of radiation pneumonia (ground-glass opacity and pulmonary infiltrate) were observed in the right lung 20 weeks after irradiation (arrow) and expanded until 28 weeks after irradiation. At 32 weeks after irradiation, findings of radiation fibrosis (consolidation with volume loss) were observed (arrowhead).

Sequential CT scans were obtained for the same three mice to follow up on the time-dependent CT changes in the lung (Figure 2). The images were reconstructed by obtaining end-expiratory images using a respiratory gating technique. At 20 weeks after irradiation, one mouse died shortly after inhalation anesthesia for a CT scan. Respiratory and circulatory failure due to oversedation and hypothermia were considered possible causes of death. The other mouse was followed until 32 weeks after irradiation. We could not observe any obvious changes in the mice until 16 weeks after irradiation. At 20 weeks, we observed patchy ground-glass opacity in the irradiated right lung. By 24 and 28 weeks, this opacity had expanded and become denser. At 32 weeks, we noted linear shadows, consolidation with lung volume loss, and compensated expansion in the non-irradiated left lung.

### 3.2. Comparison of CT Image Findings with Pathological Findings

**Figure 3:**
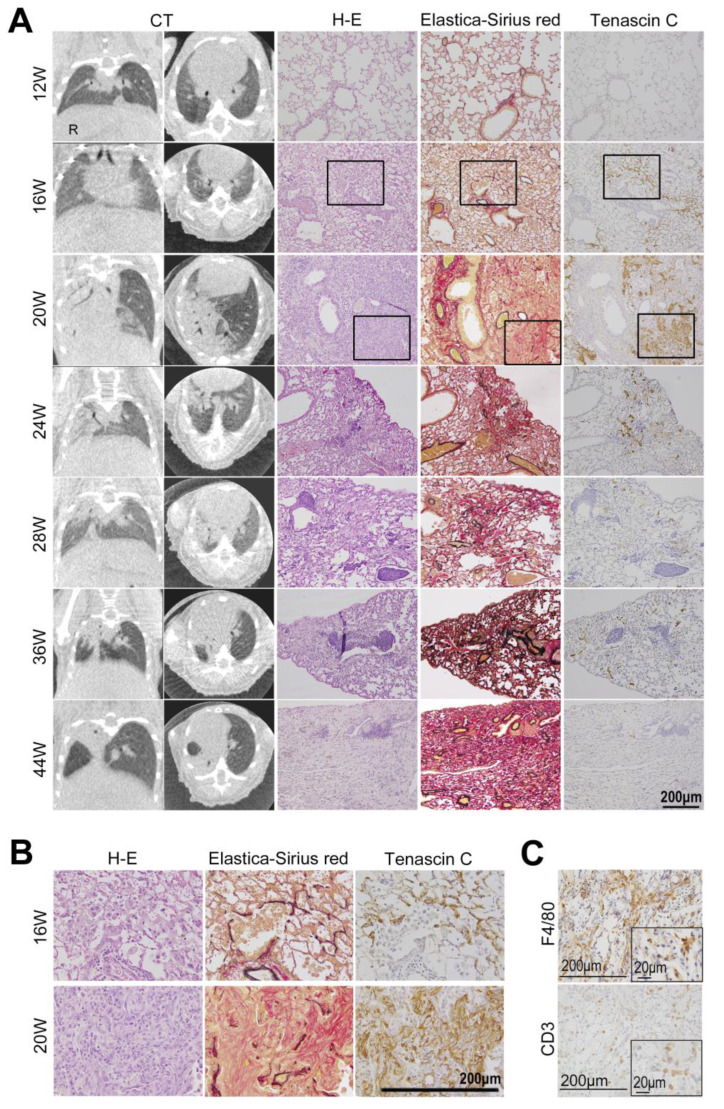
(**A**) Comparison of computed tomography (CT) image findings with pathological findings. Serial histological sections of the lung lesion were stained with hematoxylin and eosin (H-E), Elastica Sirius Red, or anti-tenascin-C. At 16 weeks, many alveolar macrophages associated with lymphocytes were found in the alveoli, whereas the CT images did not show specific findings. At 20 weeks after irradiation, the radiation pneumonia (RP) findings were confirmed by CT images and hematoxylin and eosin staining. As the CT findings changed from RP to radiation fibrosis (RF), decreasing inflammatory cells and the progression of alveoli destruction and atrophy were observed in H-E-stained lung sections. Collagen fibers appeared in the adventitia of pulmonary vessels simultaneously with RP findings on the CT images and H-E-stained sections and expanded sequentially to damaged alveoli. Tenascin-C (TNC) was expressed at 16 weeks, earlier than the changes observed with the CT images. The strong expression of TNC was found in the fibrotic area at 20 weeks and subsequently decreased. (**B**) Enlarged images of the boxed areas in (**A**). (**C**) F4/80-positive macrophages and CD3-positive T cells were seen in the inflammatory lesion at 20 weeks. The scale bar represents 200 µm or 20 µm.

We compared the CT image findings with the histological findings (Figure 3). Overall, 21 mice (three for each time point) were euthanized after CT at different time points (12, 16, 20, 24, 28, 36, and 44 weeks) after irradiation. The lungs were sampled for histological examination, and the findings were compared with those of CT. The unirradiated left lung specimens from mice were evaluated as controls. As seen in the sequentially observed individual, we observed no specific changes in the CT images at 12 and 16 weeks. At the histological level, we noted no obvious changes in structure or TNC expression at 12 weeks. However, at 16 weeks, small inflammatory lesions were observed. The deposition of TNC began to be observed in the thickened alveolar septa near the inflammatory foci (Figure 3A,B).

At 20 weeks, when clear consolidation was confirmed in the CT images, the histology of the lesions showed features of organizing pneumonia consisting of fibroblasts, macrophages, and lymphocytes, as well as loose collagen fibers (Figure 3C).

**Figure 4:**
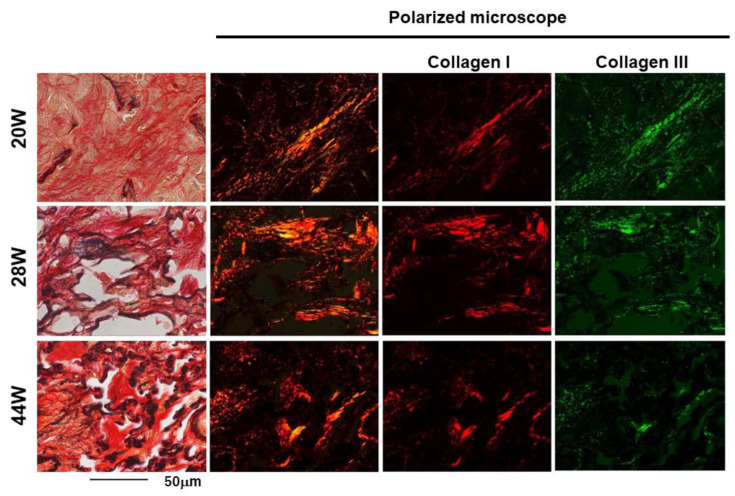
Polarized light microscopy observation of the Elastica-Sirius-Red-stained fibrotic lesion in the lung, using band-pass filters; the red fibers were composed mainly of collagen type I and the green fibers were composed mainly of collagen type III. At 20 weeks after irradiation, collagen-type-III-rich green thin fibers were widely observed in the fibrotic lesion. Subsequently, thick red collagen type I increased as fibrosis progressed from 28 to 44 weeks after irradiation. The scale bar represents 50 µm.

Collagen-type-III-rich immature collagen fibers (Figure 4) were formed in the inflammatory lesions and at the adventitia of blood vessels. The intense expression of TNC was observed in the inflammatory area, particularly in these early fibrotic lesions.

**Figure 5:**
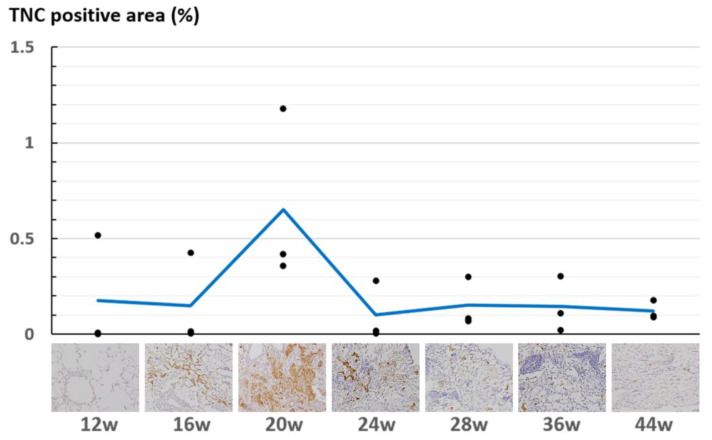
Percentage of tenascin-C-positive areas at different time points. The dots represent actual scores for tenascin-C-positive rates (n = 3 for each time point). The line graph shows the average value of each point.

At 28 to 36 weeks, when the CT findings of RP (ground-glass opacities and consolidation) were gradually replaced by the findings of RF (linear shadow, consolidation, and volume loss of the lung), the histological image showed the attenuation of inflammatory infiltrates and a marked reduction in TNC expression. The deposition of collagen-type-I-rich mature fibers increased. The quantitative analysis of the TNC-positive area showed that the TNC deposition increased during the early phase of RP, peaked at 20 weeks after irradiation, and rapidly decreased (Figure 5, Supplemental Table S1).

**Figure 6:**
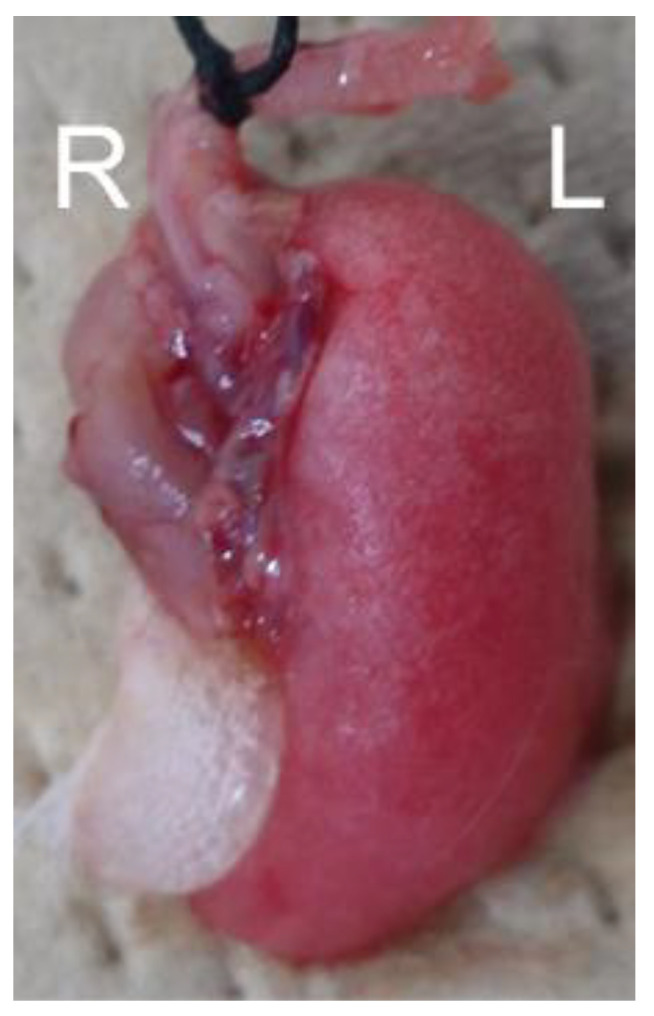
Macroscopic findings of the irradiated lung of a mouse 44 weeks after irradiation. ‘R’ represented the right lung and ‘L’ the left lung.

At 44 weeks, after the CT images showed findings of apparent RF, the macroscopic examination of the irradiated lung of the mouse showed the deformation and atrophy of the irradiated right lung, while the left lung, which was not irradiated, was normal (Figure 6). On a histological level, the inflammatory infiltrates disappeared, normal alveolar architectures were destroyed and collapsed, and collagen fibers were abundantly deposited in the lesion. TNC expression did not occur at this stage. The non-irradiated left lungs showed no changes in the CT images and pathology findings during all time periods (Supplemental Figure S1). A summary of the CT and histological findings at each period is shown in Table 1.

## 4. Discussion

Here, we developed a mouse model of inflammation/fibrosis with partial lung irradiation. RP/RF was carefully evaluated using CT images and compared with the pathological findings over a long period. Furthermore, we found that the expression of an inflammation/fibrosis marker, TNC, was upregulated during the early stage of progression in the RILD lesion before the CT findings became apparent.

### 4.1. Partial Lung Irradiation Model for Mice

We developed a mouse model of localized high-dose irradiation similar to SBRT by partially irradiating the lungs with 30 Gy/1fr. Our study examined female BALB/c mice aged 12 weeks. BALB/c mice are Th2-dominant and often used as models for fibrosis rather than for inflammation. By focusing on one sex, the total number of subjects included in the study was reduced, which is in line with current animal welfare regulations.

For RILD, human data suggest that differences occur depending on whether interstitial pneumonia or connective tissue disease is a risk factor [25]. Although women generally have a lower lung volume than men, no sex-based difference has been reported in the incidence of RILD [26]. This new model will facilitate the study of RP/RF. Only a few studies of RP/RF using mice with long-term follow-up using CT images and pathological findings have been reported. Although there have been reports of the entire or hemithorax irradiation of mice [4,27], practical SBRT only irradiates the area around the tumor. Therefore, a model mouse subjected to partial lung irradiation was needed. Some studies using small animal irradiators with small collimators, such as those with a diameter of 3–7 mm, have been reported for the study of SBRT [28,29,30,31,32]. However, the number of facilities where such equipment could be used is limited. Jin et al. prescribed a dose of 50 Gy/1fr or 90 Gy/1fr to shorten the duration of the experiment, allowing radiographic or pathologic changes to occur 1–4 weeks after irradiation [33]. Although these doses are excessive for SBRT in patients, and these radiographic changes occur earlier than those in patients subjected to SBRT, a different reaction from human RP/RF would have occurred. A preclinical study revealed several differences in fibrosis-related gene expression between lungs subjected to focal irradiation at 20 Gy and 90 Gy, implying different molecular mechanisms underlying radiation-induced lung fibrosis [33]. In this study, RF developed 28 weeks after the partial irradiation of the lungs at 30 Gy, and the onset time was similar to that in patients. In our study, fractionated irradiation was difficult due to system constraints, so we used single-dose irradiation. In human studies, single-dose irradiation to the lungs was performed at 30–34 Gy [34]. Based on these human study data, we adopted a dose of 30 Gy in one fraction. Therefore, we believe that the result is more comparable to the post-SBRT course for early-stage lung cancer in patients. This model has the potential to contribute to the optimization of treatment parameters such as the radiation dose, frequency, and irradiation area in SBRT, as well as to the elucidation of the mechanisms of RILD. Moreover, this model might be used in the future to evaluate the association between immune checkpoint inhibitors, molecularly targeted drugs, lung infections, and RILD. If a biomarker for RILD is identified, the measurement of blood concentrations using ELISA could potentially be applied to evaluate RILD in humans. The key benefit of discovering a biomarker for RILD is identifying severe cases early, potentially improving the treatment outcomes.

### 4.2. Sequential Changes in CT Images after Irradiation

We obtained sequential CT scans for the same individual in Group 1 from 12 weeks up to 32 weeks after irradiation. At 20 weeks after irradiation, patchy ground-glass opacity appeared. Subsequently, the ground-glass-like lesion gradually expanded and progressed to consolidation at around 24 to 28 weeks, followed by the development of atelectasis at 32 weeks. These time-dependent changes in the CT imaging were also confirmed by the Group 2 experiments, and the images were similar to those after SBRT for human lungs [1,35,36]. Previous mouse experiments have reported a similar sequential change in the CT findings of RP/RF. However, they appeared slightly earlier [2,5,6,7,33], possibly due to differences in the mouse strain, the irradiation field, and the irradiation dose used in the experiments.

### 4.3. Comparison of CT Images and Histological Changes

We compared the CT image findings with the histopathological findings at each stage. Early inflammatory findings were microscopically observed at 16 weeks before the CT image findings of RP became detectable. Our current immunohistological analysis cannot explain why inflammation starts as late as 16 weeks after irradiation.

The prominent proliferative changes in the lung tissue sections at 20 weeks after irradiation could reflect the “patchy ground-glass opacity” in CT imaging.

The “linear shadowing, consolidation, and loss of lung volume” at a later stage indicate progression from RP to RF. These changes could correspond to the histological findings, such as the decrease in inflammatory cells, the formation of mature collagen fibers, and the progression of destruction and atrophy. These pathological findings were similar to those of RF in humans [8,37].

Notably, collagen fiber formation started from the adventitia of pulmonary vessels in the early stage of RP and subsequently expanded to damaged alveoli. Microvascular damage is known as one of the causes of radiation-induced lung injury. Microvascular damage increases the vascular permeability and decreases the microvascular network, which leads to ischemia [8,10,37,38]. Choi et al. found that collagen deposition began around blood vessels in the early phase of RILD after irradiation in C57BL/6 mice and demonstrated that vascular hypoxia triggered endothelial-to-mesenchymal transition, leading to chronic lung fibrosis [28].

Furthermore, polarized light microscopy revealed that collagen-type-III-rich immature fibers were primarily observed in the early phase of fibrosis. Subsequently, collagen-type-I-rich thick mature fibers increased as fibrosis progressed. The change from thin, immature collagen fibers to thick, mature collagen fibers was presumed to cause RF, including contractive changes and the deviation of lung structures observed on the CT images. Immature type III collagen fibers were primarily observed at 20 weeks. This early fibrotic lesion might be reversible by anti-inflammatory drugs. The findings may also indicate the optimal time for surgical intervention in cases requiring surgery after radiotherapy. However, further elucidation of the mechanisms of fibrosis will improve radiation-induced fibrosis in the lungs and other organs, including skin and muscle induration, hollow organ stenosis, and the delayed healing and dehiscence of postoperative wounds.

### 4.4. TNC Is Upregulated at the Early Stage of Radiation Pneumonitis

Here, we found that TNC was upregulated in the early phase of RP and could be involved in profibrotic activity. Notably, the expression of TNC in the histological section of the lung lesion was detectable before the appearance of the RP findings on the CT images, suggesting that TNC could be an early marker for RP. In the quantitative evaluation of the deposition of TNC, the amount did not increase at 16 weeks but a peak was observed at 20 weeks, consistent with the pathological findings. As shown in Figure 3, the CT images of mice evaluated at 20 weeks showed more extensive RP than those of the other mice, supporting the results of the quantitative evaluation of TNC.

In this study, the ratio of the TNC-positive area to the entire lung section area was quantified. Several other quantification methods were considered. One method was to measure the TNC-positive rate in a randomly selected field of view under a microscope. In this method, there was a risk of underestimating the TNC positivity rate if lung tissue outside the irradiated area was selected. The other method of selecting regions with strong TNC expression might result in bias.

TNC is deposited in the extracellular spaces in inflammatory lesions; however, it is also released into the circulating blood. Many studies have demonstrated the clinical utility of serum TNC levels as a marker for tissue damage, inflammation, and fibrosis and a tissue remodeling marker in various diseases, including cardiovascular disease [18].

Recently, we reported a lung cancer case in which the serum level of TNC was elevated after radiotherapy [22]. The patient underwent preoperative chemoradiotherapy with a dose fraction of 40 Gy/20fr. At the end of RT and 4 weeks after RT, the serum levels of TNC were elevated compared to the pre-RT levels. At 1 week after surgery (13 weeks after RT), serum TNC decreased to the pre-RT level. A chest CT scan was obtained 2 days before surgery (13 weeks after RT), and it revealed RP findings within the irradiation field. The patient underwent surgery 13 weeks after irradiation, and TNC expression was confirmed in surgically resected lung tissue in the region where RP occurred. Postoperative pathological specimens showed the thickening of the alveolar walls. Inflammatory cells, such as macrophages, were observed at the site of radiation injury. In the alveoli, a fibro-inflammatory bud (Masson body) was found. Elastica Sirius Red staining showed fibrosis in the interstitium, and immunostaining showed multiple α-smooth-muscle-actin-positive cells in the fibrotic lesion. The expression of TNC was also found in the interstitium. The human data also showed that the serum TNC was elevated before the appearance of RP on CT imaging and pathologically confirmed TNC deposition. These results indicated that TNC was associated with the early phase of RILD in humans, which is supported by the present mouse experiment. Treatment for RILD involves the initiation of steroids [9]. A delay in this initiation could lead to serious symptoms. The decision to initiate steroids for RILD is typically based on clinical symptoms (cough, fever, difficulty breathing) and CT imaging. Nevertheless, if TNC proves to be a useful biomarker for RILD and can be confirmed prior to the change in the CT image, the early initiation of steroids might be possible. In addition, the mechanism of RILD is still unclear, especially regarding the mechanism of RF; if TNC is involved in the occurrence of RILD, it is believed to contribute to the elucidation of this mechanism.

### 4.5. Limitations

This study had some limitations. Firstly, the relationship between the irradiation dose and lung volume could not be evaluated. In the thoracic region, the irradiation dose and volume of the lung (e.g., V20 and mean lung dose) are associated with the risk of developing RILD. Because the irradiation setup in our facility was not equipped with cone-beam CT, the relationship between the irradiation dose/volume and severity of RILD could not be analyzed. Second, we were unable to perform this experiment in carcinoma-bearing mice. In the carcinoma-bearing state, different molecular mechanisms may occur in inflammation and fibrosis compared to the healthy state. Third, while this study has the potential to contribute to the understanding of the mechanisms of RILD, there is a possibility that the findings in mice might differ from those in humans.

## 5. Conclusions

We developed a new mouse model of RILD via partial lung irradiation and performed long-term imaging and a pathological evaluation. Furthermore, we evaluated the relationship between TNC expression and RILD using this mouse model and suggested that TNC is a useful inflammatory marker preceding fibrosis. It might be useful in identifying patients at a high risk of developing RILD and providing appropriate care at an early stage.

## Figures and Tables

**Table 1:** Summary of computed tomography and histological findings at each period.

Post Irradiation		12 w	16 w	20 w	24 w	28 w		36 w	44 w
Computed Tomography		−	−	Radiation pneumonia				Radiation fibrsis	
	
Histology	Inflammation	−	+	+ + +	+ +	±		−	−
	Fibrosis	−	−	+	+	+ +		+ + +	+ + +
	Tenascin-C	−	+	+ + +	+ +	±		−	−

## Data Availability

The datasets generated and/or analyzed during the current study are included in this published article.

## Supplementary Materials

The following supporting information can be downloaded at: https://www.mdpi.com/article/10.3390/cimb46090575/s1, Supplemental Figure S1, Supplemental Table S1.
