# Supplementary material for: Expression of Tenascin-C Is Upregulated in the Early Stages of Radiation Pneumonitis/Fibrosis in a Novel Mouse Model
Source: Curr Issues Mol Biol. 2024 Sep 1;46(9):9674–85. doi: 10.3390/cimb46090575 (PMC11430349; doi:10.3390/cimb46090575)
Supplement: Supplementary file 1 [file cimb-46-00575-s001.zip › Supplemental Table S1.pdf]

Table S1. The quantitative analysis of the proportion of TNC-positive area in the lung area

	TNC Area (μm^2)	Lung Area (μm^2)	TNC/Lung Ratio	Average TNC/Lung Ratio
12W	2721.99000	42266277.21100	0.00006	
	1402.13600	67527580.47700	0.00002	0.00176
	319770.75000	61691661.56000	0.00518	
16W	6481.31200	40264449.15400	0.00016	
	3555.28100	62958479.19600	0.00006	0.00149
	359164.21200	84256976.16800	0.00426	
20W	308360.55800	26148608.93400	0.01179	
	178090.31500	42481515.81300	0.00419	0.00652
	237189.78200	66591186.68300	0.00356	
24W	3274.63400	58718932.71000	0.00006	
	199357.43900	71397501.57500	0.00279	0.00101
	8645.79500	49885941.90000	0.00017	
28W	128931.64200	43136826.91100	0.00299	
	42820.41800	51977092.30000	0.00082	0.00150
	42228.74900	60612803.65400	0.00070	
36W	37415.96400	34653317.32500	0.00108	
	115562.00300	38276722.03600	0.00302	0.00144
	12134.45700	54578918.20500	0.00022	
44W	57125.12300	64117684.68900	0.00089	
	88331.41100	49902866.31500	0.00177	0.00120
	31250.52000	32822351.22100	0.00095	
