# Supplementary figures and images for: Expression of Tenascin-C Is Upregulated in the Early Stages of Radiation Pneumonitis/Fibrosis in a Novel Mouse Model
Source: Curr Issues Mol Biol. 2024 Sep 1;46(9):9674–85. doi: 10.3390/cimb46090575 (PMC11430349; doi:10.3390/cimb46090575)

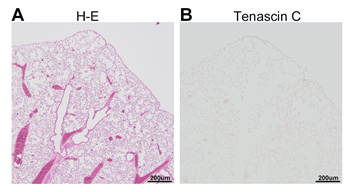

Supplement: Supplementary file 1 [file cimb-46-00575-s001.zip › Fig S1.tif]
